# Supplementary material for: Mapping Pediatric Seasonal Influenza Vaccine Safety and Immunogenicity Evidence: A Systematic Review of Clinical Trials
Source: Vaccines (Basel). 2025 Dec 26;14(1):32. doi: 10.3390/vaccines14010032 (PMC12846517; doi:10.3390/vaccines14010032)
Supplement: Supplementary file 1 [file vaccines-14-00032-s001.zip › vaccines-4028539-supplementary.pdf]

Table S1 *– Data Extraction Pilot Form*

DATA FIELD	DESCRIPTION
**Study ID**	Random unique identifier assigned to each study
**Citation**	Full bibliographic citation
**Phase**	Phase of clinical trial (I, II, III, IV, or not specified)
**Year**	Year study began enrollment
**Location**	Country or countries where the study was conducted
**Vaccination Used**	Classification of vaccinations used (subunit or sub-viral)
**Vaccine Sub-classification**	Sub-unit or split-virion
**Valency**	Trivalent or quadrivalent
**HA Content**	Hemagglutinin (HA) content in micrograms
**Vaccine Season Year(s)**	Vaccine strain formulation based on WHO recommendations
**Vaccine Season Hemisphere**	Northern or Southern based on WHO recommendations
**Vaccine Route of Administration**	Intramuscular (IM)
**Dosages Tested**	Dose amounts and number of doses administered
**Second Dose Timing**	Days after first dose the second dose was administered
**Adjuvant or Preservative Used**	Adjuvants (MF59 or Aluminum Phosphate) or preservatives (Thiomersal) in vaccine formulation
**Manufacturing Details**	Chicken egg or mammalian cell based
**Ages**	Age range studied (6 - 35 MO, 3 - 8 YO)
**Mean Age per Sample Group**	Mean age in either months of years per sample group
**Sex**	Sex distribution of study participants per sample group
**Total Sample Size**	Total number of participants for outcome of interest
**PPP (Per-Protocol Population)**	Number of participants analyzed under PP (n)
**Immediate LOB**	Immediate length of observation for safety outcomes after vaccine administration (e.g., 30 minutes)
**Total LOB**	Total length of observation after last dose safety outcomes were assessed (e.g., 6 months)
**Solicited Events LOB**	Total length of observation for solicited events
**Dose Safety Outcomes Applicable to**	First or second dose outcomes
**Method of Safety Outcome Collection**	Method utilized to record AEs
**Randomization**	Whether the study was randomized
**Blinding**	Type of blinding used (e.g., single, double, open-label)
**Baseline Status**	Influenza vaccine-primed or naïve population
**% AEs (Local)**	Percent adverse events recorded for local events pooled such as redness, swelling, or pain
**% AEs (Systemic)**	Percent adverse events recorded for systemic events pooled such as fever, cough, headache, nausea, fatigue, malaise, drowsiness
**% AEs (Total)**	Percent adverse events recorded for all events pooled
**% Solicited AEs**	Percent solicited adverse events as defined per studies
**% Unsolicited AEs**	Percent unsolicited adverse events as defined per studies
**% SAEs**	Percent severe adverse events
**SAEs Related to Vaccine**	Did study personnel determine that the severe adverse event was related to the vaccine

**SAE Details**	Details of vaccine-related severe events
**Overall Conclusion**	Any findings related to safety or immunogenicity relevant to the review question

Table S2*- Risk of Bias In Randomized Trials (RoB 2)*

**Reference**	Bias arising from the randomization process	Bias due to deviations from intended interventions	Bias due to missing outcome data	Bias in measurement of the outcome	Bias in selection of the reported result	Overall RoB
Chen, 2023 [14]	+	+	+	+	-	-
Claeys, 2018 [25]	+	+	+	+	+	+
Cruz-Valdez, 2018 [26]	+	+	+	+	+	+
Diallo, 2018 [32]	+	+	-	+	+	-
Halasa, 2015 [33]	+	+	+	+	-	-
Hu, 2020 [15]	+	+	+	+	+	+
Kothari, 2024 [22]	-	+	+	+	-	-
Langley, 2015 [23]	+	+	+	+	-	-
Ojeda, 2020 [27]	-	+	+	+	-	-
Pepin, 2019 [24]	+	+	-	+	-	-
Sarkar, 2021 [21]	-	+	+	+	-	-
Wang, 2024 [17]	-	+	-	+	+	-
Zhang, 2022 [19]	+	+	+	+	-	-

+	Low risk of bias
-	Some concerns
!	High risk of bias

***Table S3 -** Risk of Bias In Non-Randomized Studies (ROBINS-I V2)*

**Reference**	Bias due to confounding	Bias in classification of interventions	Bias due to selection of participants	Bias due to deviations from intended interventions	Bias due to missing data	Bias in measurement outcomes	Bias in selection of the reported results	Overall RoB
Agarkhedkar, 2019 [20]	x	+	-	+	+	-	x	x
Carmona Martinez, 2014 [28]	x	-	-	+	-	-	x	x
Chang, 2020 [29]	x	+	-	+	+	-	x	x
Dhamayanti, 2020 [30]	x	+	-	+	+	-	x	x
Mo, 2017 [16]	x	+	-	+	+	-	x	x
Soedjatmiko, 2018 [31]	x	+	-	+	+	-	x	x
Dhamayanti, 2020 [30]	x	+	-	+	+	-	x	x
Wen, 2025 [18]	+	+	-	+	+	-	-	+

+	Low
-	Moderate
x	Serious
!	Critical
